# Comparative Analysis of Total and Size-Fractionated Chlorophyll *a* in the Yellow Sea and Western Pacific

**DOI:** 10.3389/fmicb.2022.903159

**Published:** 2022-05-06

**Authors:** Yuqiu Wei, Zhengguo Cui, Xingzhou Wang, Guangliang Teng, Keming Qu, Jun Sun

**Affiliations:** ^1^Key Laboratory of Sustainable Development of Marine Fisheries, Ministry of Agriculture and Rural Affairs, Yellow Sea Fisheries Research Institute, Chinese Academy of Fishery Sciences, Qingdao, China; ^2^Laboratory for Marine Fisheries Science and Food Production Processes, Pilot National Laboratory for Marine Science and Technology (Qingdao), Qingdao, China; ^3^Research Centre for Indian Ocean Ecosystem, Tianjin University of Science and Technology, Tianjin, China; ^4^State Key Laboratory of Biogeology and Environmental Geology, China University of Geosciences, Wuhan, China

**Keywords:** phytoplankton, chlorophyll a, size-fractionated filtration, Yellow Sea, Western Pacific

## Abstract

Measurements of different size-fractionated chlorophyll *a* concentrations (Chl *a*) of phytoplankton assemblages *in situ* are vital for advancing our understanding of the phytoplankton size structure and thus the marine biogeochemical cycle. In the present study, we thus made a comparative analysis of total and size-fractionated Chl *a* in the Yellow Sea (YS) and Western Pacific (WP). Our results suggest that the total Chl *a* was highly variable in the YS (averaging ~1.02 μg L^−1^) and was generally 3–4-fold more than that in the WP (averaging ~0.30 μg L^−1^). The pico-sized Chl *a* had a significant contribution to total Chl *a* in the WP (range 75–88%), while the average contributions of the nano-sized and pico-sized Chl *a* to total Chl *a* in the YS were 47 and 38%, respectively, suggesting that a majority of the total Chl *a* in the YS was associated with nano- and picophytoplankton. Moreover, we applied the generalized additive models (GAMs) to explore the relationships between the total Chl *a* and that contained in each of the three size classes. These GAMs relationships suggested a continuum from picophytoplankton dominated waters to large phytoplankton (cells> 2 μm) domination with increasing Chl *a*. Finally, we made a comparison of the total Chl *a* obtained with GF/F filters and that measured from size-fractionated filtration and revealed that their corresponding concentrations are in good agreement, indicating the size-fractionated filtration had no effect on total Chl *a* determination.

## Introduction

As a ubiquitous photosynthetic pigment in phytoplanktonic species and also a readily available ocean color product, the chlorophyll *a* (Chl *a*) is generally thought to be the most widely used proxy of biological indicators, such as total phytoplankton biomass and primary productivity (Falkowski and Kiefer, 1985; Behrenfeld and Boss, 2006). At present, the Chl *a* concentration can be estimated using satellite remote sensing, *in situ* with fluorometers, or measured on filtered discrete samples though pigment or fluorometric analyses and so forth (Welschmeyer, 1994; O'Reilly et al., 1998). Whatman CF/F glass fiber filters (which have a nominal pore size of 0.7 μm and a median retention size of 0.2 μm) or 0.2-μm Nuclepore membrane filters have traditionally been used for the filtration of those discrete samples to concentrate planktonic organisms (Prepas et al., 1988; Chavez et al., 1995). However, after the discovery of photoautotrophs smaller than 2–3 μm (called picophytoplankton), such as picocyanobacteria and picoeukaryotes, the essential role of the size structure of phytoplankton in the food webs and marine ecosystems has been realized (Marañón et al., 2001; Sun et al., 2018, 2019; Richardson, 2019). Also, a suite of phytoplankton biochemical functions is controlled by cell size, including nutrient uptake, metabolic rate, growth, and sinking rate (Moloney and Field, 1991; Finkel et al., 2010; Marañón, 2015). As such, accurate measurements of the size structure of natural phytoplankton assemblages *in situ* are vital for advancing our understanding of the marine biogeochemical cycling (Marañón et al., 2001; Sun et al., 2018, 2019; Brewin et al., 2019). Conventionally, phytoplankton size structure can be quantified by partitioning total Chl *a* into three typical size classes, that is, pico- (<2 μm), nano- (2–20 μm), and micro-phytoplankton (>20 μm) (Sieburth et al., 1978). However, in biological oceanography, Whatman GF/F and 0.2-μm Nuclepore membrane filters are not adequate for the determination of size-based Chl *a* concentrations, much less identifying the different size types of phytoplankton. Consequently, size-fractionated filtration associated with membrane filters of varied composition has been developed in order to retain different size-structure Chl *a* contents.

Over the last decades, measurements of size-fractionated Chl *a* are of particular significance to the biogeochemical community and are therefore used extensively in biological oceanographic research. For instance, size-fractionated Chl *a* approach has complemented the flaw of remote-sensing algorithms with respect to the estimates of size-fractionated biomass and primary production (Varela et al., 2002; Kameda and Ishizaka, 2005). Meanwhile, measurements of size-fractionated Chl *a* have also been used to estimate the export production (Guidi et al., 2009) or used for the validation of size-based food web models and marine biogeochemical models (Marañón et al., 2001). Size fractionations have the advantage of making a explicit partition of the size structure of phytoplankton assemblages. However, this approach involving filters of different sizes (e.g., 0.2, 2 and 20 μm) poses a problem in that planktonic organisms may break apart during the continual filtration process, ultimately resulting in a certain portion of cellular Chl *a* passing through the filters into seawater (Brewin et al., 2014). On the contrary, the size-fractionated filtration is also time-consuming and thus probably alters the Chl *a* concentration, as Chl *a* fluorescence is particularly vulnerable to continuous light exposure. Such a filtration step is often taken for granted as being a relatively conservative step in an extraction procedure (Knefelkamp et al., 2007); however, its impact on the results may be underestimated. Considering the potential influence of size-fractionated filtration on the Chl *a* determination, the main question we wish to address in this paper is that: Is total Chl *a* concentration measured from size-fractionated filtration approximately equal to that obtained with Whatman GF/F filters?

Many comparisons between different filters in terms of Chl *a* retention have been carried out in previous studies. For example, both Chavez et al. (1995) and Moran et al. (1999) have compared Chl *a* measurements obtained from field samples using Whatman GF/F and Nuclepore 0.2-μm filters and verified no significant difference between the total Chl *a* concentrations estimated from these two filters. Analogously, size-fractionated Chl *a* for three size classes (i.e., < 2 μm, 2–20 μm, and > 20 μm) estimated from measurements of size-fractionated filtration and HPLC analysis have also been compared quantitatively, but there are significant biases between these two methods, with HPLC analysis underestimating picoplankton Chl *a* obviously (Brewin et al., 2014). In addition, the relationship between total Chl *a* and that contained in each of the three size classes has been studied relying on a three-component model (Brewin et al., 2010, 2019). To date, however, little is known about the degree to which size-fractionated filtration impact Chl *a* determination. In the present work, we thus make use of an expanded dataset collected from coastal waters of the Yellow Sea and from oligotrophic waters of the Western Pacific, consisting of measurements of total Chl *a* and size-fractionated Chl *a*, to quantify the relationship between total Chl *a* obtained with GF/F filters and that measured from size-fractionated filtration. More importantly, the total and size-fractionated Chl *a* data in these two distinct regions of the ocean have rarely been compared.

## Materials and Methods

### Study Area and Water Sampling

**Figure 1:**
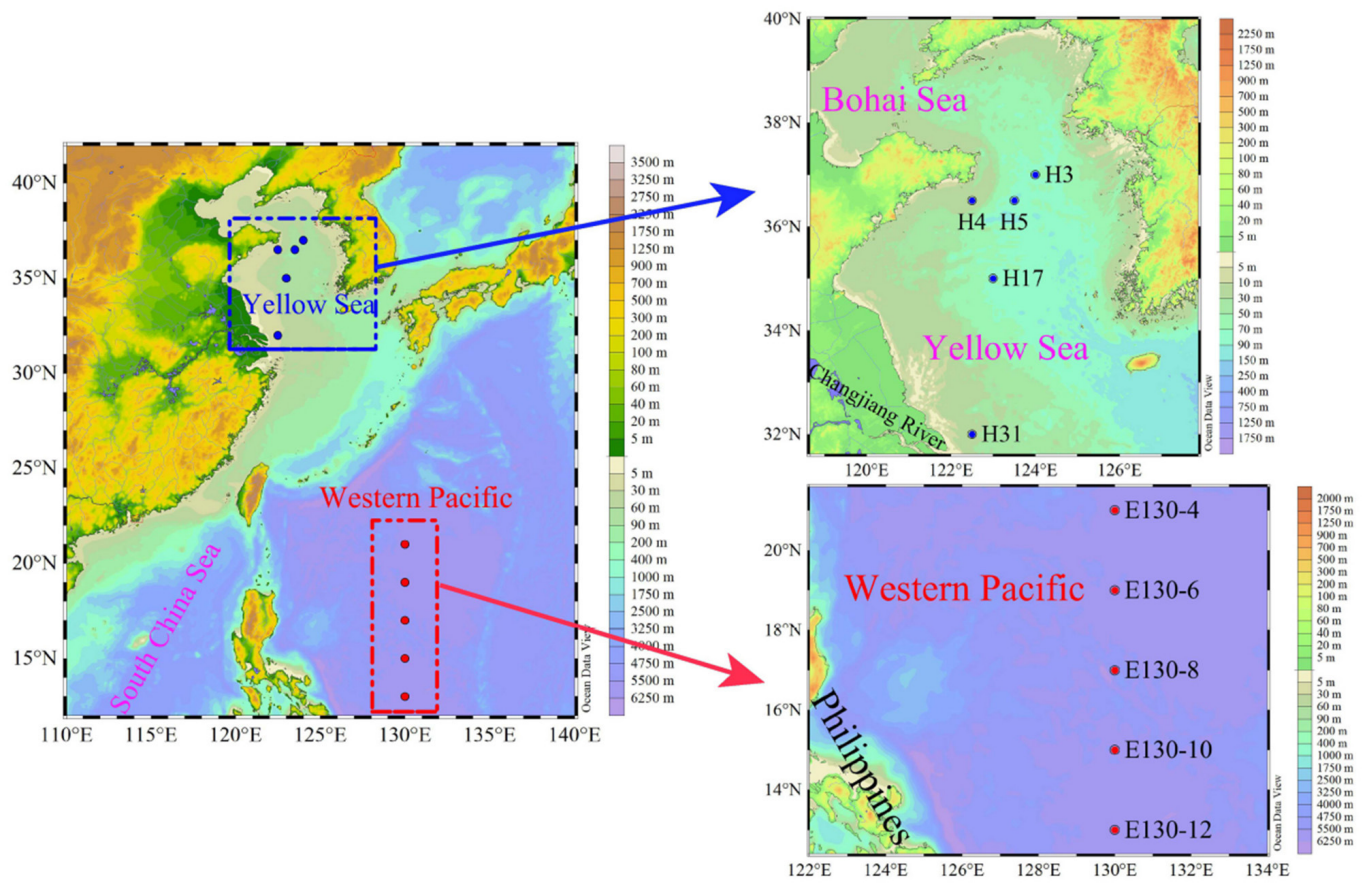
Study area and sampling stations in the Yellow Sea (blue dots) and the Western Pacific (red dots).

Results presented here encompass data from two oceanographic cruises located in the Yellow Sea, China (YS; 32–37°N, 122–124°E), and the Western Pacific (WP; 13–21°N, 130°E), covering 10 multiple spatial stations that employ very similar sampling strategies (Figure 1). The cruise for YS took place from September to October 2021 aboard R/V *Hailan* 101, while the cruise for WP was undertaken from October to November 2018 aboard R/V *Kexue* 3. Thus, a variety of oceanographic conditions and habitats, for example, the high-biomass coastal YS (Huo et al., 2020) and the nutrient-depleted WP affected by the Kuroshio (Yasuda, 2003), were sampled during these two cruises. Water sampling and parallel measurements of temperature and salinity were performed using 12-L Niskin bottles equipped with a Sea-Bird CTD (conductivity, temperature, and depth) rosette sampler (SBE 19 Plus). Seawater samples for the Chl *a* analysis were taken at up to 3 depths in the YS and 6 depths within the upper 200 m in the WP, respectively (Table 1). In total, 180 Chl *a* samples (comprising total and size-fractionated Chl *a*) were available, 60 from the YS and 120 from the WP, respectively. Both total and size-fractionated Chl *a* were measured on water collected from the same bottles, and all samples were collected during daylight hours (9:00–16:00 local time).

**Table 1:** Information of sampling stations and depths for the Chl *a* analysis during the two cruises.

**Yellow Sea**				**Western Pacific**			
**Station**	**Latitude (**°**E)**	**Longitude (**°**N)**	**Sampling depth (m)**	**Station**	**Latitude (**°**E)**	**Longitude (**°**N)**	**Sampling depth (m)**
H3	124	37	2, 29, 71	E130–4	130	21	5, 25, 50, 82, 150, 200
H4	122.5	36.5	2, 8, 17	E130–6	130	19	5, 25, 50, 93, 150, 200
H5	123.5	36.5	2, 32, 71	E130–8	130	17	5, 25, 50, 100, 150, 200
H17	123	35	2, 33, 67	E130–10	130	15	5, 25, 50, 120, 150, 200
H31	122.5	32	2, 12, 24	E130–12	130	13	5, 25, 50, 115, 150, 200

### Total and Size-Fractionated Chl *a* Analyses

The size-fractionated filtration method for measuring Chl *a* concentration in each size class involves filtering water through different filters with decreasing pore sizes. Polycarbonate (PC) membrane filters typically used for the size-fractionated filtration have a higher precision in pore size, as they are manufactured with a laser (Brewin et al., 2014). In this study, for each water sample collected from the YS and WP, ~1,000 mL of seawater was filtered sequentially through 20-μm nylon membrane, and 2- and 0.2-μm PC membrane filters (47 mm; Merck Millipore Ltd.) under low-vacuum pressure (<0.04 MPa). In addition, we particularly defined the Chl *a* concentration in each size class that we used throughout the article so that our intended meaning was more clear. Unless otherwise noted, in this study, we used the terms pico-sized Chl *a*, nano-sized Chl *a*, and micro-sized Chl *a* for the size ranges of 0.2 to 2 μm, 2 to 20 μm, and > 20 μm, respectively (Sieburth et al., 1978). Thereafter, the total Chl *a* content (called “total size-fractionated Chl *a* concentration”) was estimated from the sum of the three size classes for each sample.

Although the minimum pore size of PC filters (i.e., 0.2 μm) used above is lower than that of the GF/F filters (nominal size ~0.7 μm), no significant difference has been found for the Chl *a* retention capability between these two filters, because the GF/F filter has a median retention size (i.e., effective pore size) of ~0.2 μm. In particular, the GF/F filters have long been considered as a standard for phytoplankton Chl *a* determination (Chavez et al., 1995; Moran et al., 1999). During the two cruises, we thus applied the GF/F filters for the measurements of total phytoplankton Chl *a* content (referred to as “total Chl *a* concentration”). For this purpose, ~1,000 mL of seawater samples was directly filtered through the Whatman GF/F filters (47 mm; Whatman Corp.) under the same low-vacuum pressure (<0.04 MPa).

Following filtration, these Chl *a* filters were folded in quarters and stored in liquid nitrogen at −80°C until processing. Subsequently, pigment extraction was made by submerging the filters in 90% acetone for 24 h at 4°C. After removal of the filters, extracted pigments were then determined using a CE Turner Designs Fluorometer following the standard method of Welschmeyer (1994).

### Statistical Tests

**Figure 2:**
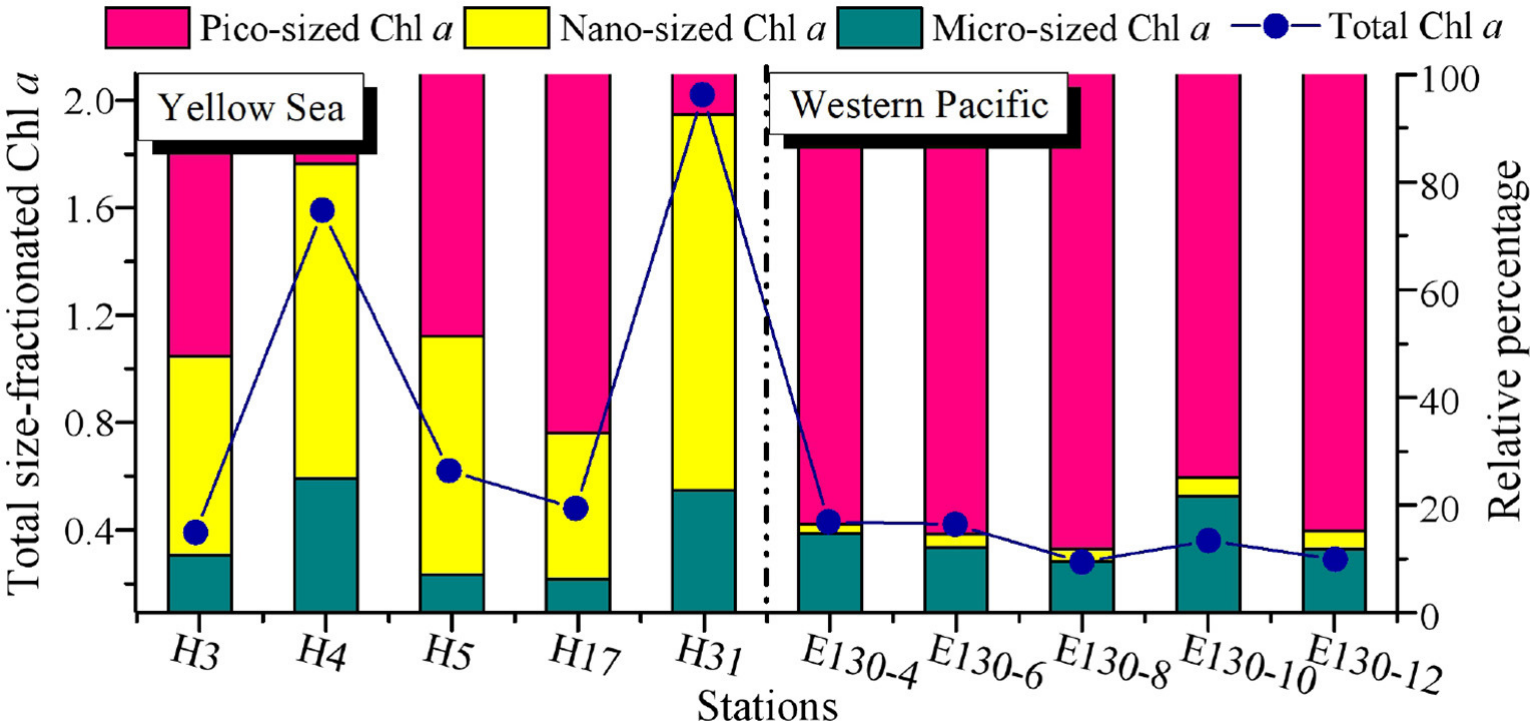
Spatial variations of total size-fractionated Chl *a* concentrations (μg L^−1^) and the relative proportion (%) for each size class among sampling stations in the Yellow Sea and the Western Pacific. Blue dots and lines denote the total size-fractionated Chl *a* concentrations, which were the sum of the three size classes. Note that all the size-fractionated Chl *a* data were expressed as depth-weighted averages, corresponding to the *y*-axis on the left.

Average data were given as values ± standard deviation (i.e., SD). The non-linear regression models (i.e., Gauss and Lorentz models; Origin v8.5) and *t*-test (Prism v8.3) were used to plot the fitting curves and thus to explore the vertical trends of the size-fractionated Chl *a*. Spearman's correlation analysis (*r* and *p*-values; SPSS, v25) and linear regression (R^2^; Origin v8.5) were used to evaluate the relationship between total Chl *a* obtained with GF/F filters and that measured from size-fractionated filtration (Wei et al., 2020). Furthermore, to establish the relationship between total Chl *a* and that in three size classes (Brewin et al., 2014, 2019), we applied the generalized additive models (GAMs) that were performed using R software in version 4.0.2. The GAMs were fitted with the mgcv package (GAMs with GCV smoothness estimation). All statistical significance levels were set to *p* < 0.05. Unless otherwise stated, the total and size-fractionated Chl *a* concentrations used for presenting spatial variation or for comparison among stations were expressed as depth-weighted averages (See Figure 2 below), which were calculated by dividing the trapezoidal integration of measured values for each variable by the maximum sampling depth (Crosbie and Furnas, 2001). The depth-weighted equation was calculated as follows:


$$\begin{array}{l} \text{Ch}{\text{l}}_{a}=\left[\overset{n}{\underset{1}{\sum }}\frac{\left(\text{Ch}{\text{l}}_{ai}+\text{Ch}{\text{l}}_{ai+1}\right)}{2}\times \left(D_{i+1}-D_{i}\right)\right]/\left(D_{MSL}-D_{S}\right) \end{array}$$

where “Chl *a*” is the depth-weighted average (μg L^−1^) over the sampling water column; “Chl *a*_*i*_” is the Chl *a* concentration (μg L^−1^) at sampling layer *i*; “*n*” is the number of sampling layers in the YS (*n* = 3) and WP (*n* = 6), and “*D*_*i*_” is the depth at sampling layer *i* (m); “*D*_MSL_” and “*D*_S_” are the depths of maximum sampling layer and the surface sampling depth, respectively (Table 1).

## Results and Discussion

### Total and Size-Fractionated Chl *a* Concentrations

#### Horizontal Distribution

There was a wide range of variability in the magnitude of total size-fractionated Chl *a* concentrations (referred to as “Chl *a*_>0.2μ*m*_”) among sampling stations across the YS and WP, but significant contribution of pico-sized Chl *a* (i.e., 0.2–2-μm size class) to Chl *a*_>0.2μ*m*_ was observed in the WP (Figure 2). The Chl *a*_>0.2μ*m*_ was highly variable in the YS, ranging from 0.39 to 2.02 μg L^−1^, with an average value (±SD) of 1.02 ± 0.73 μg L^−1^ (Table 2). In particular, the station H31 had the highest Chl *a*_>0.2μ*m*_ than other stations in the YS, while the Chl *a*_>0.2μ*m*_ was relatively low at station H3. The station H31 was observed near the Changjiang River estuary (Figure 1), which receives strong influences from nutrient-rich fresh water of the Changjiang River (Liu et al., 2016), thereby resulting in seasonally and spatially high phytoplankton Chl *a*, biomass, and primary production (Gong et al., 2003; Guo et al., 2014). Differently, the station H3 was dramatically influenced by the Yellow Sea Cold Water Mass (YSCWM, T < 10°C; Fu et al., 2018), which forms large temperature difference between the surface and bottom, reaching up to ~1°C (Lee et al., 2016). Conceivably, the relatively low Chl *a*_>0.2μ*m*_ at station H3 may be controlled by the cold water in the YSCWM. On the contrary, this result may be due to the YSCWM nutrient-depleted surface water, with N and P concentrations near the analytical detection limit (Fu et al., 2018), although the YSCWM represents a large nutrient reservoir in the bottom layer of the YS. Indeed, the Chl *a*_>0.2μ*m*_ in the YS was strongly related to temperature (*p* < 0.05) and dissolved inorganic N, P, and Si (*p* < 0.05) in our correlation analysis (Supplementary Figure 1A), suggesting that temperature and nutrients were the two key factors regulating the variation of Chl *a*_>0.2μ*m*_ in the YS. On the contrary, the Chl *a*_>0.2μ*m*_ in the WP was lower and less variable, averaging 0.30 ± 0.06 μg L^−1^ (range 0.24–0.36 μg L^−1^; Figure 2 and Table 2). The mean Chl *a*_>0.2μ*m*_ in the WP was generally 3–4-fold lower than that in the YS. Similarly, the Chl *a*_>0.2μ*m*_ was positively correlated with nutrients (*p* < 0.05) in the WP (Supplementary Figure 1B), indicating that the observed low Chl *a*_>0.2μ*m*_ in the WP was largely caused by nutrient limitation. We thus suggested that the dynamic of Chl *a*_>0.2μ*m*_ in the WP could be reasonably driven by the Kuroshio current (Wei et al., 2020), since it is characterized by substantially higher temperature and salinity, and yet very low nutrient concentrations (Yasuda, 2003).

**Table 2:** Depth-weighted averages of total and size-fractionated Chl *a* concentrations (μg L^−1^) among sampling stations in the Yellow Sea and the Western Pacific.

**Cruise**	**Station**	**Micro-sized Chl *a***	**Nano-sized Chl *a***	**Pico-sized Chl *a***	***Chl a*** **_>0.2μ*m*_**
Yellow Sea	H3	0.04	0.14	0.21	0.39
	H4	0.40	0.93	0.26	1.59
	H5	0.04	0.27	0.30	0.62
	H17	0.03	0.13	0.32	0.48
	H31	0.46	1.41	0.15	2.02
Western Pacific	E130–4	0.06	0.01	0.36	0.43
	E130–6	0.05	0.01	0.36	0.42
	E130–8	0.03	0.01	0.24	0.28
	E130–10	0.08	0.01	0.27	0.36
	E130–12	0.03	0.01	0.25	0.29

The Chl *a* concentration in particles > 20 μm (i.e., micro-sized Chl *a*) was relatively low in the YS (average 0.19 ± 0.21 μg L^−1^) and WP (average 0.05 ± 0.02 μg L^−1^) (Table 2), and thus, the contribution of micro-sized Chl *a* to the Chl *a*_>0.2μ*m*_ across both regions was only between 6 and 25% and averaged 14 ± 7% (Figure 2). Likewise, the contribution of 2–20 μm Chl *a* concentration (i.e., nano-sized Chl *a*) to the Chl *a*_>0.2μ*m*_ was comparatively low in the WP, averaging at 3 ± 1% and ranging from 2 to 4%. Our understanding of why the Chl *a* concentration is high or low in the ocean could be guided by analyzing the phytoplankton community data. Hence, these low contributions were not surprising given that the dominants of phytoplankton in the oligotrophic WP are not micro/nano-sized species, where large diatoms only represent on average ~15% of total C biomass compared to small picophytoplankton (~47%) (Wei et al., 2020). In comparison, the nano-sized Chl *a* at the coastal YS was between 27 and 70% of the Chl *a*_>0.2μ*m*_ and averaged 47 ± 17%, which was consistent with previously reported contribution of the nano-sized Chl *a* in the Garolim and Asan bays of the YS (range 17–71% and average 40 ± 14%; Lee et al., 2020). Furthermore, Soria-Píriz et al. (2017) have also shown that nanoplankton was the dominant fraction of Chl *a* in the coastal estuary (e.g., the Gulf of Nicoya) representing 51–78% of total Chl *a*. In our previous study, we indeed found that the phytoplankton community in the YS basin was mainly dominated by the nano-sized *Paralia sulcata, Thalassiosira angulata, Thalassiosira excentricus*, and *Skeletonema* cf. *costatum* (Wei et al., 2017). It is noteworthy that the station H31 situated near the Changjiang River estuary also had a greater nano-sized Chl *a* concentration (1.41 μg L^−1^, ~70% of the Chl *a*_>0.2μ*m*_), which may be associated with the dominant species *Skeletonema* cf. *costatum* therein (Guo et al., 2014).

Across the WP, the Chl *a* concentration in < 2-μm size class (i.e., pico-sized Chl *a*) was higher but less variable than that in other size classes among sampling stations (range 0.24–0.36 μg L^−1^, average ~0.29 μg L^−1^; Figure 2 and Table 2). The average contribution of pico-sized Chl *a* to the Chl *a*_>0.2μ*m*_ in the WP was close to 83 ± 9% (range 75–88%), indicating that a majority of the Chl *a*_>0.2μ*m*_ was in picoplankton size fraction, and the Chl *a*_>0.2μ*m*_ variability was largely driven by the pico-sized Chl *a*. This significant fraction of pico-sized Chl *a* in the Chl *a*_>0.2μ*m*_ is broadly consistent with previous findings in open-ocean ecosystems (Fiala et al., 1998; Froneman et al., 2001; Morán et al., 2004; Richardson, 2019). In the oligotrophic NE Atlantic, for example, most of the total Chl *a* was present in small cells (< 2 μm), and picoplanktonic Chl *a* contributed approximately 75% to total values (Morán et al., 2004). Similarly, our previous studies in the oligotrophic eastern Indian Ocean and South China Sea have also revealed that the average concentration of pico-sized Chl *a* accounted for ~50% of the total Chl *a* (Wei et al., 2020). Collectively, these high contributions could be attributable to the fact that picophytoplankton have contributed a significant proportion of the phytoplankton community in oligotrophic ecosystems, for example, the WP. In contrast to the WP, the pico-sized Chl *a* concentrations in the YS varied from 0.15 to 0.32 μg L^−1^ with mean (±SD) of 0.26 ± 0.07 μg L^−1^, but their contributions to the Chl *a*_>0.2μ*m*_ were lower, averaging ~38% and ranging from 8 to 67% (Figure 2 and Table 2).

#### Vertical Distribution

**Figure 3:**
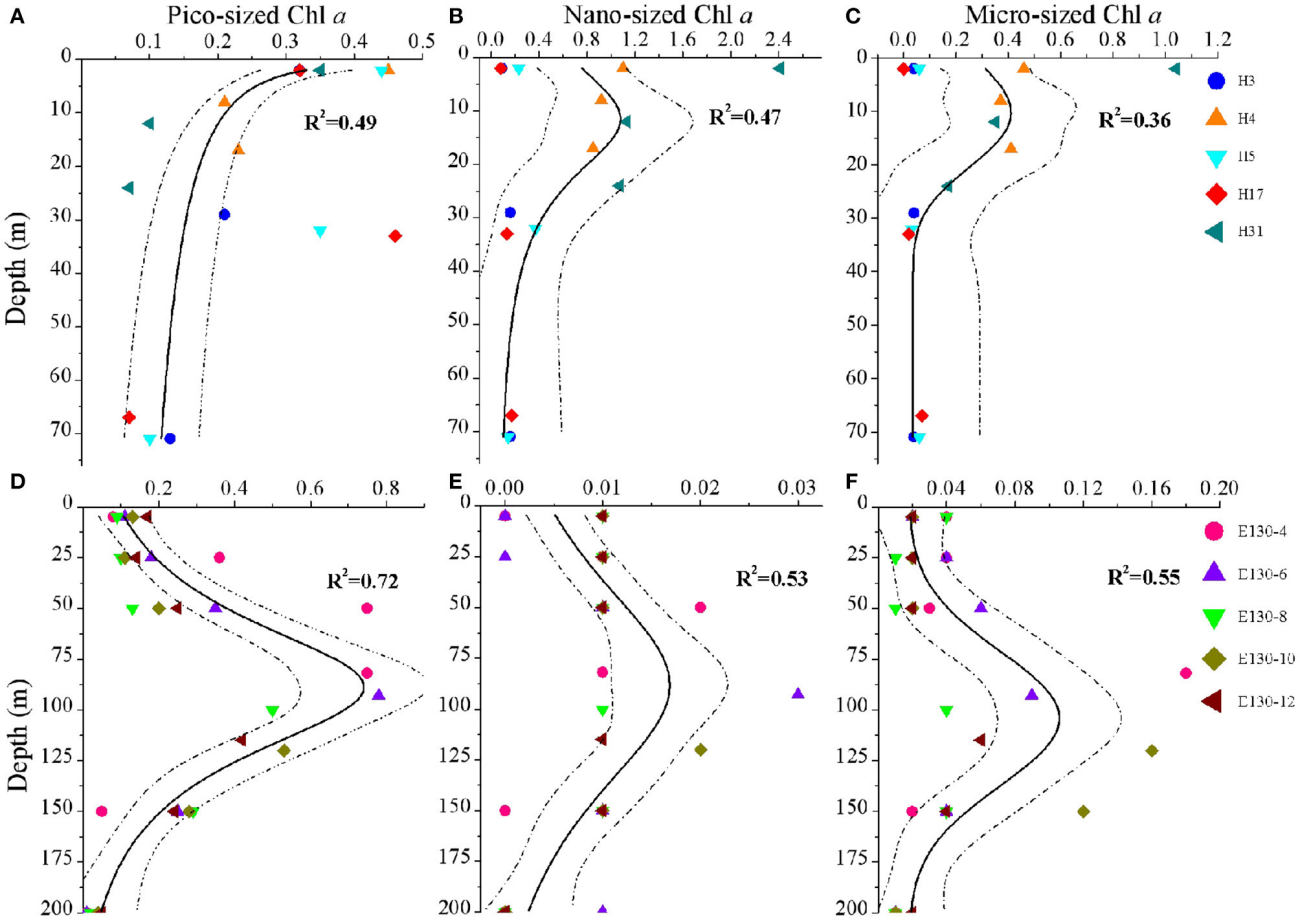
Vertical profiles for the concentrations (μg L^−1^) of **(A,D)** pico-sized Chl *a*, **(B,E)** nano-sized Chl *a*, and **(C,F)** micro-sized Chl *a* in the Yellow Sea (above) and the Western Pacific (below). Symbols and colors represent sampling stations. Solid lines indicate different curve-fitting trends for the size-fractionated Chl *a* data vs. depth, and dashed lines denote 95% confidence bands. R^2^ is the fitting variance of the non-linear regression model. Note the different scales on the *x*-axes, reflecting variability in the magnitude of the size-fractionated Chl *a* concentrations.

All data points of the size-fractionated Chl *a* concentrations against depth were analyzed to plot the fitting curves in Figure 3, and several major vertical trends were observed in the concentration of Chl *a* in each size fraction. The vertical patterns of pico-sized Chl *a*, nano-sized Chl *a*, and micro-sized Chl *a* were markedly different between the YS and the WP. Nevertheless, there were some similarities in the vertical patterns of these three size fractions collected from the same waters, especially in the WP (Figures 3D–F).

At the coastal YS, analysis of pico-sized Chl *a* profile showed that the pico-sized Chl *a* was generally highest in the surface waters and declined with depth (Figure 3A); however, for the profiles of micro-sized and nano-sized Chl *a*, they were typically low near the surface, gradually increased to a subsurface maximum at 10–15 m, and then rapidly declined to < 0.2 μg L^−1^ (Figures 3B,C). Information on the phytoplankton community structure is essential to understand the characteristics of size-fractionated Chl *a* distribution (Fu et al., 2018; Wei et al., 2020). Analysis of the abundance data of *Synechococcus* and picoeukaryotes also revealed that their total abundance was maximum at the surface and declined with depth (Supplementary Figure 2A). Therefore, the vertical variability of pico-sized Chl *a* appears to be driven largely by picophytoplankton dynamics (Supplementary Figure 3A). Our results are also in line with previous findings regarding the vertical structures of picophytoplankton abundance and pico-sized Chl *a* in the YS, for example, Fu et al. (2018) have reported that high picophytoplankton abundance occurs in the nutrient-depleted upper 30 m of the central YS area. A major mechanism has been proposed to explain the formation of this vertical pattern in the YS: active growth under an optimal combination of light and nutrients (Fu et al., 2018). Field data and experimental studies have suggested that nutrients are depleted in the surface waters of the central YS area (e.g., YSCWM), in particular, the phytoplankton growth is influenced by P limitation throughout the YS (Wei et al., 2017; Yang et al., 2018). Because of their competitive advantages in low nutrient concentrations (e.g., P) and under stratification conditions, picophytoplankton could dominate in the surface mixed layer of the YS (Supplementary Figure 2A). Light is another long-standing hypothesis and is frequently suggested to explain the vertical decrease in the phytoplankton in the euphotic zone (Guo et al., 2014; Liu et al., 2016). In our study, the pico-sized Chl *a* was significantly associated with light irradiance (Supplementary Figure 1A), indicating that light is likely responsible for the vertical decrease in the pico-sized Chl *a* in the YS. Accordingly, we concluded that the active growth of picophytoplankton under an optimal combination of nutrient and light availability was the main formation mechanism for the vertical variation of pico-sized Chl *a* in the YS. The vertical trends of micro-sized and nano-sized Chl *a* also corresponded with the vertical distribution of large phytoplankton assemblages in our previous findings in the YS (Wei et al., 2017). Compared with the surface mixed layer with high irradiance, large phytoplankton assemblages could well be acclimated to the low-light but nutrient-replete subsurface layer. Therefore, nutrient availability was the most important contributing factor to the vertical trends of micro-sized and nano-sized Chl *a* in the YS (Supplementary Figure 1A).

The major vertical trends of the three size-fractionated Chl *a* concentrations were as expected for the WP. Within the upper 200 m, their concentrations were generally 2–4-fold higher near the subsurface 100 m than in the surface layer, with a rapid decline at depths deeper than 125 m (Figures 3D–F). It has been reported that nutrients are almost depleted over the surface in the oligotrophic WP due to the water stratification, whereas nutrient supply is sufficient in the deeper layer (Ma et al., 2019). Under the influence of nutrient availability, the phytoplankton assemblages characterized by a great biomass of picophytoplankton in the WP are also lower near the surface, gradually increase to a subsurface maximum at ~100 m, and then decline rapidly from 125 to 200 m (Supplementary Figures 2B, 3B). Furthermore, phytoplankton growth is limited under conditions with sufficient nutrients below the euphotic layer, which may be reasonably controlled by light limitation as discussed above (Guo et al., 2014; Liu et al., 2016). In this study, similarly, we observed that the size-fractionated Chl *a* concentrations in the WP were positively correlated with nutrients (especially N and P; *p* < 0.05), but negatively correlated with light intensity (*p* < 0.05) (Supplementary Figure 1B), indicating nutrients and light intensity were the key factors in regulating the biogeographic variation of the size-fractionated Chl *a*. We suggested that the vertical trends for the size-fractionated Chl *a* concentrations observed in the WP were primarily attributed to the combined effects of nutrient and light availability. It is noteworthy that the vertical variation of Chl *a* in the offshore waters or the open oceans is typical of the deep chlorophyll maximum (DCM) which is formed at the thermocline layer (Gong et al., 2003; Behrenfeld and Boss, 2006; Uitz et al., 2006; Brewin et al., 2017; Sun et al., 2019). The implication is that the vertical variation of Chl *a* may be related to vertical stratification (Chen et al., 2021).

### Relationships Between Total Chl *a* and That in Three Size Classes

**Figure 4:**
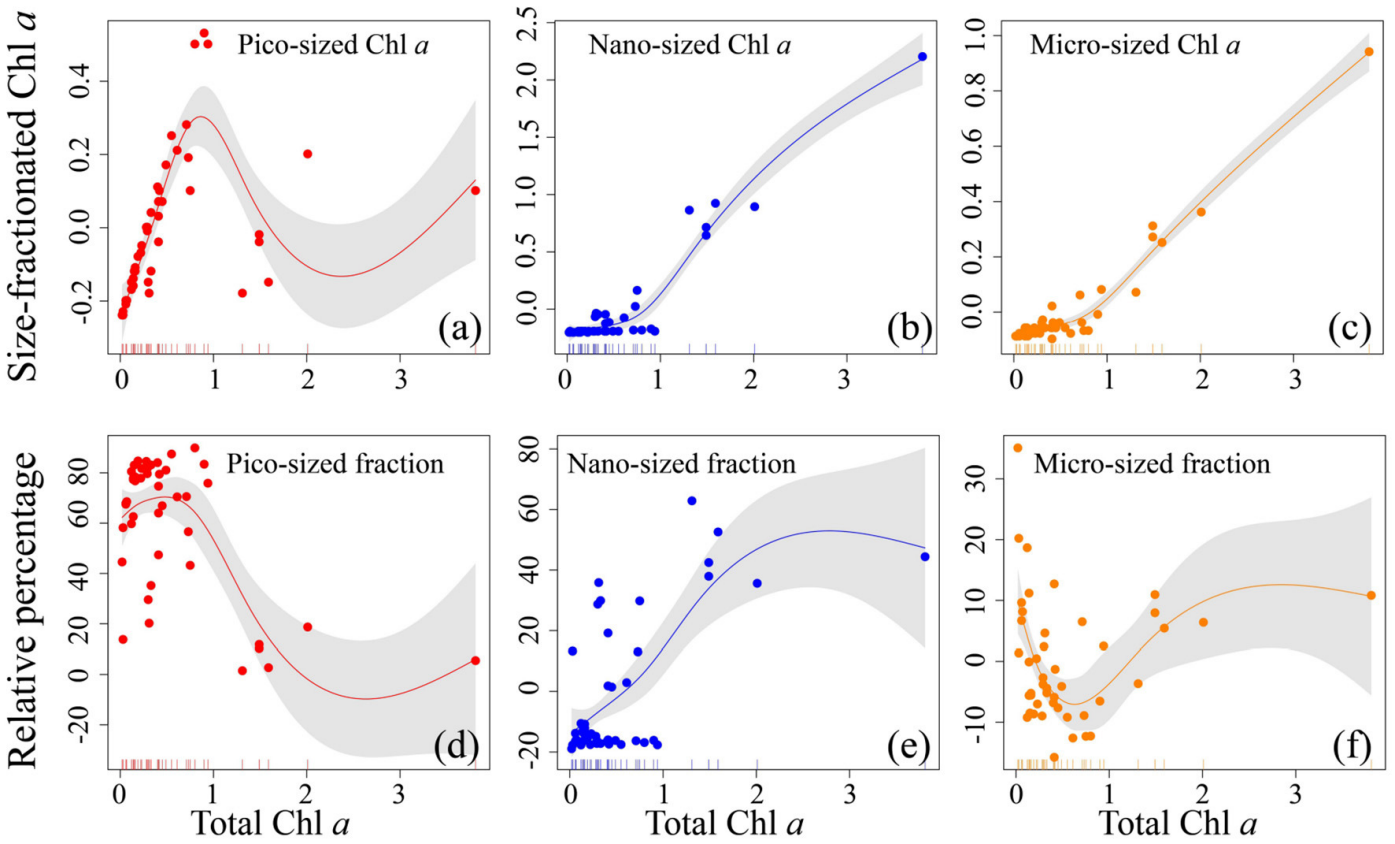
Relationship for all size-fractionated filtration data collected in the Yellow Sea and Western Pacific. Top row **(a–c)** shows the absolute size-fractionated Chl *a* concentrations (μg L^−1^), and bottom row **(d–f)** is the fractions (%) plotted as a function of total Chl *a* (μg L^−1^), with the GAMs overlain. The colored dots represent residual values (that are normalized by the GAMs) of both size-fractionated Chl *a* and relative percentages, and the inward tick marks on the horizontal axes show data distributions. The solid lines represent GAMs smoothing, and shaded areas denote 95% confidence bands.

The relationships between total Chl *a* and that contained in each of the three size classes have been studied primitively in some regions. Thereafter, these relationships have been quantified empirically, statistically, and/or mechanistically (Hirata et al., 2008), but one popular approach to modeling these relationships at present is the three-component model of Brewin et al. (2010). This model has an advantage over other empirical methods in that its variable parameters are interpretable, and the derived three-component relationship can be compared with a range of environmental factors, for instance, with temperature and light availability (Brewin et al., 2015, 2017). Moreover, the three-component model of Brewin et al. (2010) has been applied in the eastern China seas (Sun et al., 2018, 2019), such as the Bohai Sea, Yellow Sea, and East China Sea, though the Chl *a* estimations are derived from HPLC pigments. In the present study, differently, we applied the GAMs to establish the relationships between total Chl *a* and that in three size classes (Figure 4). The GAMs have distinct advantages over other conceptual or empirical models (Brewin et al., 2010; Hirata et al., 2011) as they allow non-linear functions of covariates to be included in regression equations and require an additive combination of functions of covariates, avoiding stringent restrictions imposed by parametric assumptions (Wood, 2006; Young et al., 2011). In the GAMs, the variable of interest is often smoothed using a locally weighted scatterplot smoothing that includes smoothing terms taking the form of non-parametric functions of predictors; thus, the GAMs using univariate or bivariate smoothers are a robust multivariate statistical method for large datasets and popular in marine studies (Young et al., 2011; Xiao et al., 2018). Altogether, the GAMs results below demonstrated that such established relationships could successfully capture the general changes in size-fractionated Chl *a* and fractions of total Chl *a* when plotted as a function of total Chl *a*, although the environmental variables were not incorporated into the models for reasons that our dataset was relatively small.

Across the coastal YS and the oligotrophic WP, the pico-sized Chl *a* increased drastically with increasing total Chl *a* when total Chl *a* was < ~1 μg L^−1^ (Figure 4a), indicating that small picophytoplankton dominate at low Chl *a* concentrations, a conclusion consistent with our observations that the pico-sized Chl *a* had a significant contribution to total Chl *a* (range 75–88%) and thus dominated in the oligotrophic WP (Figure 2), and previous studies that picophytoplankton dominate oligotrophic oceans, contributing > 10% of global primary productivity (Raven, 1998; Crosbie and Furnas, 2001; Flombaum et al., 2013; Visintini et al., 2021). In contrast, the pico-sized Chl *a* declined when total Chl *a* exceeded ~1 μg L^−1^, while the micro-sized and nano-sized Chl *a* increased gradually (Figures 4b,c), suggesting that large phytoplankton (cells >2 μm) dominate at high Chl *a* concentrations. This result is also in line with previous reports that the large phytoplankton primarily including diatoms and dinoflagellates are dominant in the coastal waters with relatively high Chl *a* concentrations (Guo et al., 2014; Sun et al., 2018; Xiao et al., 2018). Indeed, there is accumulating evidence that diatoms and dinoflagellates, two typical groups that form phytoplankton blooms, play key roles in marine coastal ecosystems and form the basis of many aquatic food webs (Guo et al., 2014; Spilling et al., 2018; Xiao et al., 2018; Trombetta et al., 2020). In addition, these relationships are very consistent with previous models of Brewin et al. (2010, 2019) and Sun et al. (2018, 2019) using size-fractionated Chl *a* data in the coastal Red Sea, the oligotrophic Atlantic Ocean, and the eastern China seas, with the abundance of small cells increasing to a given total Chl *a* concentration (~1 μg L^−1^), beyond which total Chl *a* increases through the addition of larger size classes of phytoplankton. This feature is also evident in the models of Uitz et al. (2006) who have suggested that an enhanced pico-sized Chl *a* near the surface is noted at the expense of the micro- and nano-sized Chl *a*. Meanwhile, our established relationships further confirmed the assumptions that the phytoplankton size structure (expressed here as size-fractionated Chl *a*) covaries with the total Chl *a* (Raimbault et al., 1988; GoeRicke, 2011). In other words, biomass is added only to the smallest size class until an upper limit to Chl *a* in this size class (called biomass quota) when phytoplankton communities are dominated by picophytoplankton and total biomass is low. For instance, the biomass quotas for the <1 <3, and <10 μm size classes are about 0.5, 1, and 2 μg L^−1^ Chl *a*, respectively (Raimbault et al., 1988). Once this quota has been reached, total Chl *a* is added to a system by the addition of larger size classes of phytoplankton. Therefore, the implication is that such relationships based on the GAMs could fit the size-fractionated Chl *a* data well in this study.

The relationships modeled to estimate fractional contributions of various size classes can offer the distinct advantage of providing more information than those that treat only the dominant class. For example, the column-integrated biomass and the vertical phytoplankton size class composition can be inferred by the Uitz et al. (2006) model that has related the Chl *a* concentration to the fractional contributions of three phytoplankton size classes (micro-, nano-, and picophytoplankton) to the total pigment. Here, a relationship was also developed to show the change in percentage contribution of the size-fractionated Chl *a* with increasing total Chl *a* (Figure 4). As anticipated, the trend of the pico-sized fraction was similar to that of the pico-sized Chl *a* observed above. The pico-sized fraction remained relatively stable increase between 0.01 and 1 μg L^−1^ total Chl *a*, whereas decreased with increasing total Chl *a* in the approximate range of ~1–3 μg L^−1^ (Figure 4d). This supports the common observation that low Chl *a* environments in oligotrophic waters are essentially dominated by picophytoplankton, where they contribute as much as ~60–90% of the total Chl *a* (Wei et al., 2020). On the contrary, the micro-sized fraction decreased with increasing total Chl *a* when total Chl *a* was low (<1 μg L^−1^), as the total Chl *a* increased beyond ~1 μg L^−1^ it began to increase (Figure 4f). This result is well-comparable to previous studies of Hirata et al. (2008), Brewin et al. (2010, 2019), and Sun et al. (2019) who have documented that microplankton begin to dominate the total population as the total Chl *a* exceeds 0.95–1.3 μg L^−1^. Moreover, there was a successive increase in the nano-sized fraction with increasing total Chl *a*, indicating that nanophytoplankton are also dominant at high Chl *a* concentrations. Collectively, these relationships between total Chl *a* and that in three size classes suggested a continuum from picophytoplankton dominated waters to micro- and nanophytoplankton domination with increasing Chl *a* (Hirata et al., 2008; Brewin et al., 2010). The implication is that our GAMs relationships not only can offer direct biological interpretation but also can be applied to a continuum of Chl *a* concentrations without having to deal with discrete trophic classes. Therefore, our GAMs relationships may be used in conjunction with algorithms designed to estimate the major variations of size-fractionated Chl *a* to improve the estimates of remotely sensed primary production (Varela et al., 2002; Kameda and Ishizaka, 2005; Hirata et al., 2008). However, as this analysis was based on a relatively small dataset (180 Chl *a* samples), we recognized that additional data were required to optimize our GAMs relationships observed here. Furthermore, additional environmental knowledge could be introduced to improve the performance of our GAMs relationships. Unfortunately, these GAMs relationships are still in their infancy for the limitation of our small dataset. Though beyond the scope of the present study, future efforts are needed in this direction.

### Does Size-Fractionated Filtration Affect Total Chl *a* Determination?

The size-fractionated filtration involving filters of different sizes leads to a series of problems in that (*i*) the filters may retain particles smaller than the nominal pore size (Prepas et al., 1988; Chavez et al., 1995; Moran et al., 1999), which is dependent on the morphology and cohesiveness of the particles, as well as on the filtered volume and the used filter types; (*ii*) some larger particles may also pass through the nominal pore size of the small filter (e.g., through overlapping holes) and be accounted for in smaller-size fractions (Brewin et al., 2014); (*iii*) the phytoplankton may break apart during the continual filtration process, ultimately resulting in a certain portion of cellular Chl *a* passing through the filters into seawater; finally, the size-fractionated filtration is time-consuming that may alter the Chl *a* fluorescence properties (Wei et al., 2019). Therefore, it is possible that the size-fractionated filtration could have an impact on the total Chl *a* determination. If this hypothesis is real, the influence could be substantially large in marine biogeochemistry, as the size-fractionated Chl *a* and phytoplankton size structure are two strong ecological descriptors of the marine biogeochemical cycling that co-vary with each other (Marañón et al., 2001; Brewin et al., 2019). On the contrary, if it is not real, the implications of our results may be minimal.

**Figure 5:**
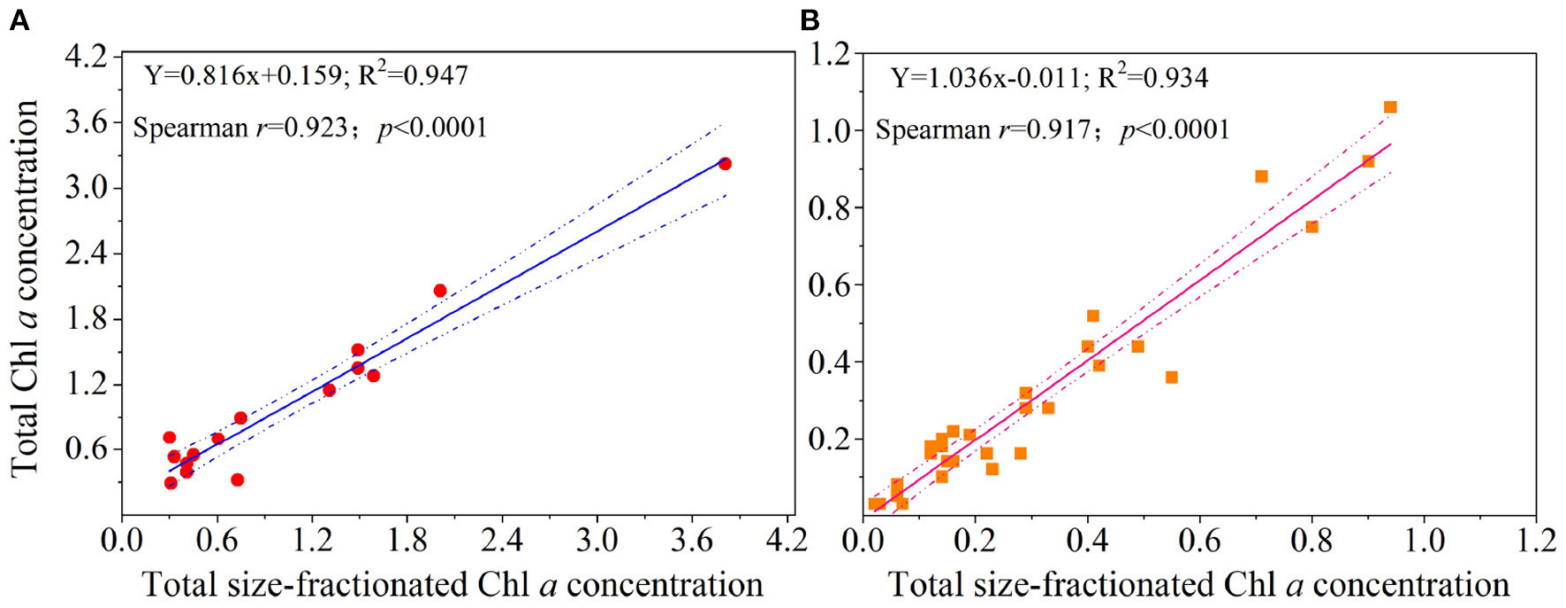
Comparisons of the estimates of total Chl *a* concentration (μg L^−1^) obtained with GF/F filters (i.e., total Chl *a* concentration) and size-fractionated PC filters (i.e., total size-fractionated Chl *a* concentration) in the **(A)** Yellow Sea and the **(B)** Western Pacific. Solid lines represent the linear regressions (Spearman's *r, p*-values, and regression variance R^2^), and dashed lines are 95% confidence bands.

At present, the effect of those factors above on measurement uncertainties is difficult to quantify, and we thus conducted a simultaneous measurement of the total Chl *a* obtained with GF/F filters and PC filters to make an accurate diagnosis of uncertainty in the size-fractionated technique. Across the YS and WP, surprisingly, corresponding concentrations of the total Chl *a* measured from GF/F filters and size-fractionated PC filters are in good agreement (R^2^> 0.93; *r* >0.91, *p* < 0.0001; Figure 5), suggesting that the hypothesis we presented above is not valid and thus the implications of our results may be minimal. Therefore, the comparison of total Chl *a* between GF/F filters and the sum of size-fractionated PC filters is promising for people who are using either method. Based on the raw data (Figure 5), however, our linear regressions also revealed that the overall total Chl *a* obtained with GF/F filters was slightly higher than that measured from PC filters in the YS, and an opposite trend was observed in the WP. This result reflected the fact that the size-fractionated filtration may be by no means exact, although it is encouraging to observe significant agreement between the two approaches (*p* < 0.0001; Figure 5). In accord with this logic, we speculated that there would be large or small discrepancies in each size-fractionated Chl *a* by using filters with different materials as a result of inaccuracies in pore sizes, filter clogging, and/or cell breakage as discussed above. Also, another possible reason for these potential discrepancies may be the difference in two study areas. Nevertheless, we could not address this hypothesis at present due to the technological limitations. With this in mind, future efforts should be further focused toward quantifying the uncertainty in size-fractionated filtration, possibly through simultaneous measurements made by multiple types of *in situ* methods.

## Data Availability Statement

The original contributions presented in the study are included in the article/Supplementary Material, further inquiries can be directed to the corresponding authors.

## Author Contributions

JS and ZC contributed to the theoretical designs. YW, XW, and GT performed all experiments. YW and KQ analyzed all data. YW wrote the manuscript with the help of all authors. All authors contributed to the final version of the manuscript.

## Funding

This work was financially supported by the Project funded by China Postdoctoral Science Foundation (2021M703590), the Shandong Postdoctoral Innovation Talent Support Program (SDBX2021014), the National Nature Science Foundation of China grants (41876134), the Central Public-Interest Scientific Institution Basal Research Fund, YSFRI, CAFS (20603022022010), and the Qingdao Postdoctoral Applied Research Project, and the State Key Laboratory of Biogeology and Environmental Geology, China University of Geosciences (GKZ21Y645).

## Conflict of Interest

The authors declare that the research was conducted in the absence of any commercial or financial relationships that could be construed as a potential conflict of interest.

## Publisher's Note

All claims expressed in this article are solely those of the authors and do not necessarily represent those of their affiliated organizations, or those of the publisher, the editors and the reviewers. Any product that may be evaluated in this article, or claim that may be made by its manufacturer, is not guaranteed or endorsed by the publisher.
