# Supplementary material for: Comparative Analysis of Total and Size-Fractionated Chlorophyll a in the Yellow Sea and Western Pacific
Source: Front Microbiol. 2022 May 6;13:903159. doi: 10.3389/fmicb.2022.903159 (PMC9120765; doi:10.3389/fmicb.2022.903159)
Supplement: Supplementary file 1 [file Data_Sheet_1.docx]

**Supporting Information**

**Fig. S1**


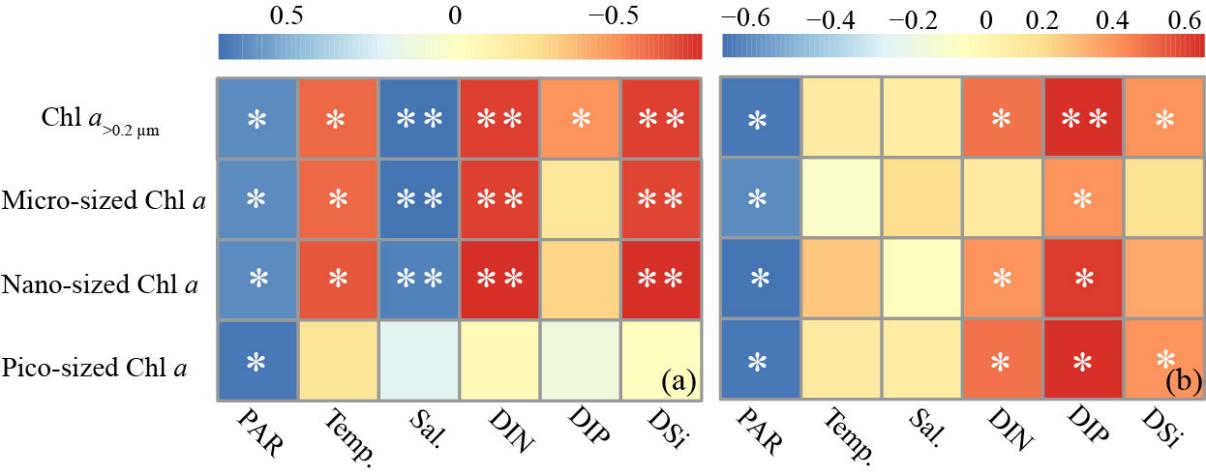


Fig. S1 Relationships between size-fractionated Chl *a* concentrations (i.e., micro-sized Chl *a*, nano-sized Chl *a*, and pico-sized Chl *a*) and various environmental factors (i.e., PAR, temperature, salinity, DIN, DIP, and DSi) in (a) the Yellow Sea and (b) the Western Pacific. Spearman correlation analysis (*r* and *p* values; SPSS, v25) was used to examine the significant relationship between size-fractionated Chl *a* concentrations and various environmental factors. Analysis results were subsequently visualized based on “pheatmap” package in R software (v 3.6.1). Spearman correlation coefficients ranged from negative to positive and are indicated by color intensity changing from dark blue to red, respectively. ** *p* < 0.01; * *p* < 0.05 (two-tailed). Chl *a*_>0.2 µm_ is total size-fractionated Chl *a* concentrations, PAR (photosynthetically available radiation) was measured using an underwater PAR sensor (RBR, XRX-620). Temp. is temperature, and Sal. is salinity. Nutrient samples were filtered through 0.45 µm cellulose acetate membrane filters (47 mm), stored in 150 mL PE vials and immediately frozen at -20 ℃ for further analysis. After returning to the laboratory, the nutrients including DIN (dissolved inorganic nitrogen, the sum of NH_4_^+^, NO3^-^, and NO2^-^), DIP (dissolved inorganic phosphorus), and DSi (dissolved inorganic silicate), were measured on a Technicon AA3 AutoAnalyzer (Bran + Luebbe) (Brzezinski & Nelson, 1986; Collos et al., 1992; Chen et al., 2021).

**Fig. S2**

Fig. S2 Vertical profiles for the total picophytoplankton abundance in (a) the Yellow Sea and (b) the Western Pacific. Symbols and colors represent different sampling areas. Solid lines indicate different curve-fitting trends for the data of total picophytoplankton abundance versus depth, and dashed lines denote 95% confidence bands. R^2^ is the fitting variance of the nonlinear regression model. Seawater samples for picophytoplankton analysis (~2 mL) were initially incubated in the dark (10-15 min) at room temperature, and then fixed with paraformaldehyde (1% final concentration) and immediately freeze-trapped in -80℃ liquid nitrogen (Jiao et al., 2005). Sub-samples with a total volume of 198 µL were analyzed using a flow cytometer (BD FACSAria^TM^ III) equipped with 488 and 640 nm laser beams. Approximately 10000-100000 events were detected on this flow cytometer. *Prochlorococcus*, *Synechococcus*, and picoeukaryotes were classified and enumerated by their different fluorescence signals and scatter properties. Meanwhile, 2 µm yellow-green fluorescent beads (Polysciences) were added as an internal standard. Note that the total picophytoplankton abundance in the Western Pacific was the sum of *Prochlorococcus*, *Synechococcus* and picoeukaryotes, while that in the Yellow Sea was the sum of *Synechococcus* and picoeukaryotes, as the *Prochlorococcus* was undetectable therein.

**Fig. S3**


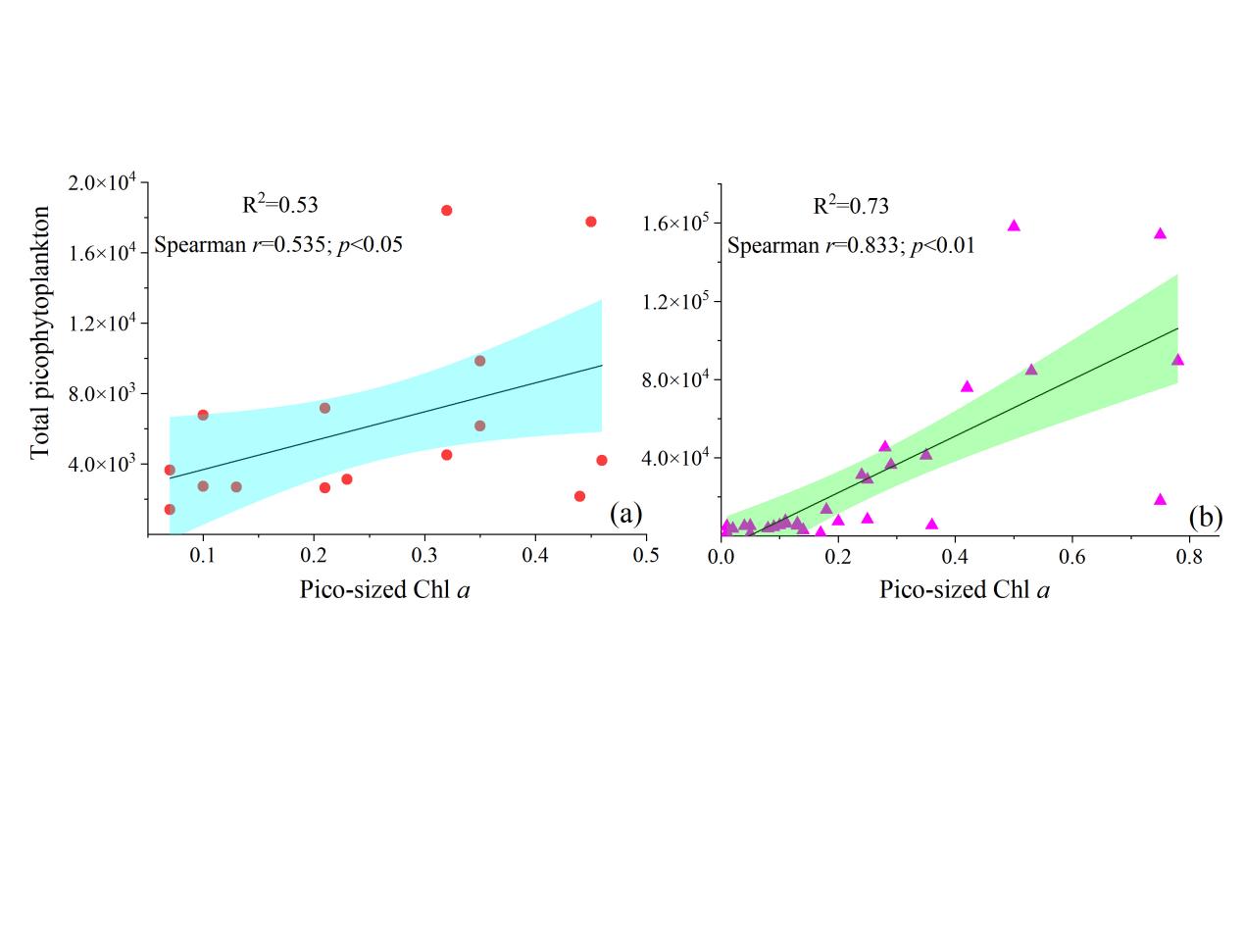


Fig. S3 Significant relationships between total picophytoplankton abundance (cells mL^-1^) and pico-sized Chl *a* concentrations (µg L^-1^) in the (a) Yellow Sea and the (b) Western Pacific. Solid lines represent the linear regressions (Spearman *r*, *p* values and regression variance R^2^) and shaded areas are 95% confidence bands.

**References**

Brzezinski, M. A., & Nelson, D. M. (1986). A solvent extraction method for the colorimetric determination of nanomolar concentrations of silicic acid in seawater. Marine Chemistry, 19(2), 139-151.

Collos, Y., Yin, K., & Harrison, P. J. (1992). A note of caution on reduction conditions when using the cadmium-copper column for nitrate determinations in aquatic environments of varying salinities. Marine chemistry, 38(3-4), 325-329.

Chen, Z., Sun, J., Gu, T., Zhang, G., & Wei, Y. (2021). Nutrient ratios driven by vertical stratification regulate phytoplankton community structure in the oligotrophic western Pacific Ocean. Ocean Science, 17(6), 1775-1789.

Jiao, N., Yang, Y., Hong, N., Ma, Y., Harada, S., Koshikawa, H., & Watanabe, M. (2005). Dynamics of autotrophic picoplankton and heterotrophic bacteria in the East China Sea. Continental Shelf Research, 25(10), 1265-1279.
